# Role of In Vitro Tests in the Characterisation of Locally Applied, Locally Acting Drugs in the Throat: Application to Flurbiprofen

**DOI:** 10.3390/pharmaceutics16101261

**Published:** 2024-09-27

**Authors:** Vit Perlik, Hafsa Ali, Jean M. Cardot, Anuradha Kulasekaran

**Affiliations:** 1 Institute of Pharmacology, First Faculty of Medicine, Charles University, Albertov 4, 128 00 Prague, Czech Republic; vitperlik@gmail.com; 2 Reckitt Healthcare Limited, 103-105 Bath Road, Slough SL1 3UH, UK; hafsa.ali@reckitt.com; 3 SAS BORVO, 18 Av de Charade, 63122 Ceyrat, France; jean-michel.cardot@wanadoo.fr

**Keywords:** locally applied locally acting, LALA, GIT, IVPT, IVRT, lozenge, throat, flurbiprofen, pharmacokinetics

## Abstract

**Background/Objectives:** For locally applied, locally acting generic drug products, comparison to an originator product based on systemic exposure is usually not feasible due to low plasma concentrations and inadequate reflection of local exposure at the site of action. Where a validated PD model exists, a comparative clinical study can be performed in healthy subjects; where no surrogate endpoint is available, patients with the relevant indication need to be enrolled, with all the associated factors which could result in lack of sensitivity. Even though the need for alternative in vitro approaches has been acknowledged by both industry and regulatory bodies, the complexity of in vivo drug delivery processes makes the development of guidance documents particularly difficult. Our objective was to present in vitro approaches less classically used and to address in vivo relevance of the selected tests. **Methods:** This article analyses current regulatory approaches in Europe and the U.S., and highlights the key advantages of in vitro tests in terms of their sensitivity, reliability, reproducibility and in vivo relevance using locally applied flurbiprofen in various formulations. **Results:** The in vitro esophageal retention (IVOR) model demonstrates that the first 6–10 min after application of different flurbiprofen formulations is important for their comparison and also offers the best correlation with in vivo data using the partial area under the concentration-time curves (pAUCs). Rheological evaluations further demonstrated that the mucoadhesive properties of the gel spray formulation are based on interaction with mucin. **Conclusions:** Designing a relevant in vitro test requires adequate evaluation of the complexity of the drug substance, drug product, dosing conditions and delivery processes.

## 1. Introduction

The recent European Medicines Agency (EMA) guideline on locally applied, locally acting (LALA) products in the gastrointestinal (GI) tract [1] recognises that “alternative models (including in vitro and in vivo methods) may have a higher sensitivity than traditional clinical and pharmacodynamic (PD) endpoints studies to detect possible differences between medicinal products containing the same active substances”, provided they have been proven to accurately reflect in vivo drug release and availability at the sites of action. EMA has also published a limited number of product specific guidelines. However, very few of these contain specific recommendations for the use of in vitro approaches for LALA drugs acting in the GI tract (e.g., Ferric Citrate) [2].

The US Food and Drug Administration (FDA) has published many Product Specific Bioequivalence Guidances [3] including some for LALA products, topical products, inhaled products and products acting in the GI tract. In those guidelines, under certain circumstances, comparative bioavailability can be evaluated using in vitro approaches even for drugs acting in the mouth and throat, such as chlorhexidine dental tablets [4]. The in vitro approach as an alternative to classical clinical studies for LALA drugs was also confirmed by the Federal Food, Drug, and Cosmetic Act [5,6].

In practice, as highlighted in the guidelines, for LALA drug products which are administered for an intended local or regional action in the GI tract, the assessment of bioequivalence or comparative bioavailability between different products by the area under the concentration-time curve (AUC) and the maximal systemic or local concentration (C_max_) is particularly challenging [1,7]. A comparison based on systemic exposure (i.e., blood levels) may not be feasible due to low systemic concentrations and may reflect absorption over the whole GI tract rather than local exposure at the site of action. Quantitative parameters, such as local concentrations, used for the evaluation of the availability of the active substance in the target region are often difficult to sample, or exhibit a low accuracy, and are subject to inherent in vivo variability. Valid in vitro tests could be used as a surrogate; however, relevance for the in vivo situation must be demonstrated. While this may be straightforward for products such as antacids (e.g., neutralisation test, calculation of buffer capacity) or intra-intestinal chelators (e.g., complexation test), it is more complicated for products acting locally on the throat due to the relatively short contact time.

For products applied to the throat, for example, although assessment of local drug availability is feasible in some instances, the measures of drug concentration in saliva or swabbing of tissues result in highly variable data which are influenced by a variety of factors such as individual saliva production, variability of the surface swabbed, etc., requiring complex validation and thus greatly increasing the number of subjects to be enrolled in a study. As a consequence, when standard bioequivalence studies are not feasible, comparative therapeutic equivalence studies are in principle considered necessary. Where a validated pharmacodynamic (PD) model exists, with an estimable surrogate endpoint, a comparative study can be performed in healthy subjects (e.g., vasoconstriction test for locally acting corticosteroids), or if a correlation between in vitro and in vivo has been established and validated, in vitro data could be used as in vivo surrogate [1,8]. If such a surrogate endpoint is not available, patients suffering from the index condition need to be included in a clinical study. This, in turn, is associated with a number of well-known confounding factors such as individual illness evolution, comedication, concomitant diseases, lifestyles, etc., resulting in high variability of the measured outcome data. In consequence, such studies are challenging in terms of identifying suitable therapeutic endpoints, defining clinically relevant differences, and control of confounding factors. These factors together with the inherent high inter-subject variability make these studies less suitable for comparison between different formulations either during product development or product lifecycle management [1].

The classic method of in vivo comparison of the performance of formulations such as generics is through bioequivalence (BE) studies [7]. Any in vitro surrogate used as alternative must also be able to assess the similarity of performance of the respective generic formulations; due to the sensitivity of in vitro tests, application of suitable methods is often referred to as IVBE (in vitro bioequivalence). Usually, more than one in vitro method should be combined to ensure a reliable assessment of the similarity of the formulations, as alternative models also have their specific intrinsic limitations. In the case of topical products, in vitro equivalence assessment is performed in stepwise manner starting from formulation comparison (qualitative and quantitative), followed by comparison of physicochemical characteristics. Thereafter, more complex methodologies such as rheology, in vitro release tests (IVRT) and in vitro permeation tests (IVPT) are used, as appropriate. The comprehensive evaluation of such series of assessments creates a body of evidence supporting IVBE. In the following, such a stepwise approach is presented for a LALA formulation [9].

In the case of LALA products for the mouth and throat, complexity of in vivo behavior also depends on other factors which significantly influence local availability, for example residence linked with tissue adherence of the drug at the site of action, remaining residual quantity at the surface depending of the residence time, the penetration within upper layers of the mucosa with or without reservoir effects, the surface area exposed and drug presence and effect in all layers of target tissues in vivo, etc. For all these factors, different in vitro systems exist, each with their specific strengths and weaknesses. Therefore, the selection of an appropriate set of experiments is crucial and depends on various formulation factors. Furthermore, for LALA products in the GI tract, Section 1 of the guideline [1] summarises six topics to be taken into account: the sites of action, the location of the target, the mechanism of action, the pharmacokinetic properties, the pharmaceutical form and the state of the drug substance in the dosage form.

It is well-established that, for orally administered drugs, appropriate in vitro dissolution tests may be sufficient to waive additional in vivo bioequivalence studies, especially for products for which in vivo drug release and dissolution are not considered a primary rate limiting factor for drug absorption [7,10]. In recent years, the assessment of LALA products has evolved, and replacement of classical clinical studies with alternative in vitro models aside from dissolution tests is also being considered. However, the absence of clinical studies and the choice of in vitro or ex vivo tests need to be justified [1].

As described in Perlik [11], the classical bioequivalence approach based on C_max_ and AUC parameters may be insufficiently sensitive for LALA products aimed at the throat. In contrast, the analysis of partial AUCs (pAUCs) which describe systemic exposure during the initial period after administration, i.e., up to 15 to 120 min after application, is a meaningful measure for early absorption and provides useful data that allow for the discrimination between different LALA formulations acting at this site. These authors found that pAUC values for different formulations were correlated with the clinically assessed time to onset of action (pain relief after oral application of flurbiprofen), and concluded that pAUC values are a valid surrogate of local exposure and activity at the site of action in this setting. Even though the pAUC values represented less than 5% of the total AUC, they correlated best with the onset of action, a finding which suggests that the onset of pain relief is an effect of rapid local absorption at the site of action rather than of systemic drug exposure.

The purpose of this paper is to present in vitro approaches that are less classically used. The adhesion (for assessment of in vitro release properties and presence at the site of action) was evaluated with an in vitro oesophageal retention (IVOR) test as compared to artificial membranes, while tissue penetration was compared in a model using human pharynx tissue (previously described in [12,13], and summarised in this article only for the purpose of discussion). Finally, the viscosity of these formulations was compared in classical rheological tests with and without mucin to evaluate the potential adhesive properties of the formulation. This series of tests was used for assessing the performance of a locally acting and locally applied product in the GI tract, to compare the performance across formulations with and without potential adhesive properties and explore the body of evidence addressing in vivo relevance of the selected in vitro tests. The approach is applied to flurbiprofen formulated as a lozenge, a spray solution or a spray gel.

## 2. Materials and Methods

### 2.1. Formulations

Three different pharmaceutical forms corresponding to four formulations were used in the experiments. The products were tested at the dose recommended in the current SmPC of the products [14,15]. Two products tested were commercially available products, one in the forms of spray solution (containing 8.75 mg flurbiprofen in three puffs) and another in the form of lozenge (containing 8.75 mg flurbiprofen in one lozenge). The remaining two formulations were spray gel corresponding to the above-mentioned spray solution modified by the addition of viscosifiers in various type/quantities (containing 8.75 mg flurbiprofen in 3 puffs). The spray gels were manufactured by adding 2.5% (*w*/*v*) Vivapur 591P for gel spray A and 2.3% (*w*/*v*) Vivapur 591P for gel spray B (J. Rettenmaier & Söhne GmbH, Rosenberg, Germany) and 0.2% (*w*/*v*) xanthan gum to the spray solution. The selection of the Vivapur was based on its inert nature linked with a high functionality as viscosifier and stabilizing capacity to insure optimal stable gel with difference in viscosity based on small difference in the amount of Vivapur.

All studied products were provided by Reckitt Benckiser Healthcare Limited, Hull, UK.

### 2.2. Preparation of the In Vitro Dosing

For lozenge, which is a solid dosage form, and thus not comparable directly to solutions, a solution was prepared. Depending on the experiment, different solutions were prepared taking into account the estimated physiological conditions as well as the requirements of in vitro experiments. For the purpose of the IVOR model, in which viscosity of media could be of importance, 21 mL of artificial saliva based on potassium Carbonate in a Sodium and Potassium Phosphate buffer system with the additional salts Sodium, Magnesium and Calcium chloride [16,17,18] was used for one lozenge to have a volume sufficient for the duration of the experiment and sufficient for the constant flow needed to mimic the natural saliva production during sucking of a lozenge, resulting in a nominal concentration of 0.42 mg/mL. That resulted in continuous flow using the muco-adhesion models with standardised duration time of contact (6 min at a rate of 3.5 mL/min, see Section 2.3).

The spray solution was not further modified. The dose of 8.75 mg flurbiprofen is delivered in a volume of 0.540 mL (three puffs).

### 2.3. Adhesion Tests

Various muco-adhesion studies were performed on the solubilised lozenge, the commercial spray solution and the viscous spray gels. All analyses were performed at 37 °C. Retention of test formulations was determined using a custom built IVOR model as previously described by Young and Smart [19]. A cellulose membrane soaked with artificial saliva was mounted onto a Perspex test cell (150 × 15 mm) and the retention profile samples were collected. The angle of the slope was 30° for the “Asleep” model and 45° for the “Awake” model. The test formulations were applied only on the upper zone representing the throat (initial 50 mm segment of the 150 mm total length). For the spray solution and the gel spray formulations, the samples were applied by three full depressions of the spray piston with the mass applied recorded by reverse weighing. After a 30 s hold time, the artificial saliva flushing was set at a 2 mL/min (Awake model) or 1 mL/min (Asleep model) flow rate. The lozenge solution was applied at a flow rate of 3.5 mL/min for 6 min with the slope at a 45° incline (a total volume of 21 mL containing 8.75 mg flurbiprofen) to simulate the sucking of the lozenge, followed by a flow of 2 mL/min of blank saliva for 30 min (Awake model). In the Asleep model, after the application of the lozenge solution, the slope was adjusted from 45° to a 30° incline and the artificial saliva flow rate was then started at 1 mL/min for up to 30 min. The time of 6 min of sucking is close to what is observed in vivo for lozenges [8].

The eluent was collected at intervals of 2, 4, 6, 8, 10, 15, 20, 25 and 30 min for flurbiprofen analysis. After 30 min, the artificial saliva flow was stopped, and the surface of the slope and the membrane was rinsed with 10 mL deionised water which was collected and analysed separately to assess the remaining quantity of flurbiprofen at the end of the experiment.

All samples collected during the experiment were assayed using a validated HPLC method with LLOQ of 0.045 µg/mL and the release percentage calculated according to the total dose applied [20].

Finally, the retention/release in vitro data obtained in these experiments expressed as AUCs (up to10 min) were compared with the respective in vivo PK ratios for pAUCs of different pharmaceutical forms reported previously [11] in order to establish in vivo relationship of the IVRT test, using a linear regression model.

### 2.4. Rheological Tests

A set of rheological tests including the Shear Rate Sweep profiles were performed on mixtures of samples of spray formulation and gel spray formulation with mucin, mimicking more closely the in vivo context. Mucin solutions were prepared by dispersion into deionised water, corresponding to an intermediate concentration of 10%. After pH adjustment to 6.2 using sodium hydroxide solution 0.5 M, the concentration was adjusted to the final value of 6%. Mixtures of mucin solution with the tested formulations (1:1 by weight) were prepared. All samples were prepared in duplicate and subjected to rheological testing after overnight rest. Experiments were performed at 37 °C on a Discovery Series Hybrid Rheometer (DH2R, TA Instruments, New Castle, DE, USA) using a 60 mm aluminium plate at a gap size of 200 µm. A solvent trap cover was employed to minimise drying of the sample at the exposed edges of the plates.

### 2.5. Ex Vivo and In Vivo Human Data Correlation

In order to establish the relevance of IVOR, the in vitro release data expressed as AUCs (0 up to 30 min) for the different pharmaceutical forms were compared with previously reported clinical AUCs for the first 15 min after administration (0–15 min) and the first 30 min after administration (0–30 min) [11]. Clinically, these AUCs correlated with the onset of action after administration (i.e., the time to clinical onset of pain relief), and may serve therefore as a surrogate of local tissue absorption of flurbiprofen. For interstudy comparison the spray formulation was used as a reference, and in vitro AUCs as well as clinical AUCs were expressed as ratios to the reference. Clinical and in vitro AUCs ratio were plotted, and their relationship assessed by linear regression.

## 3. Results

### 3.1. Adhesion Tests

**Figure 1:**
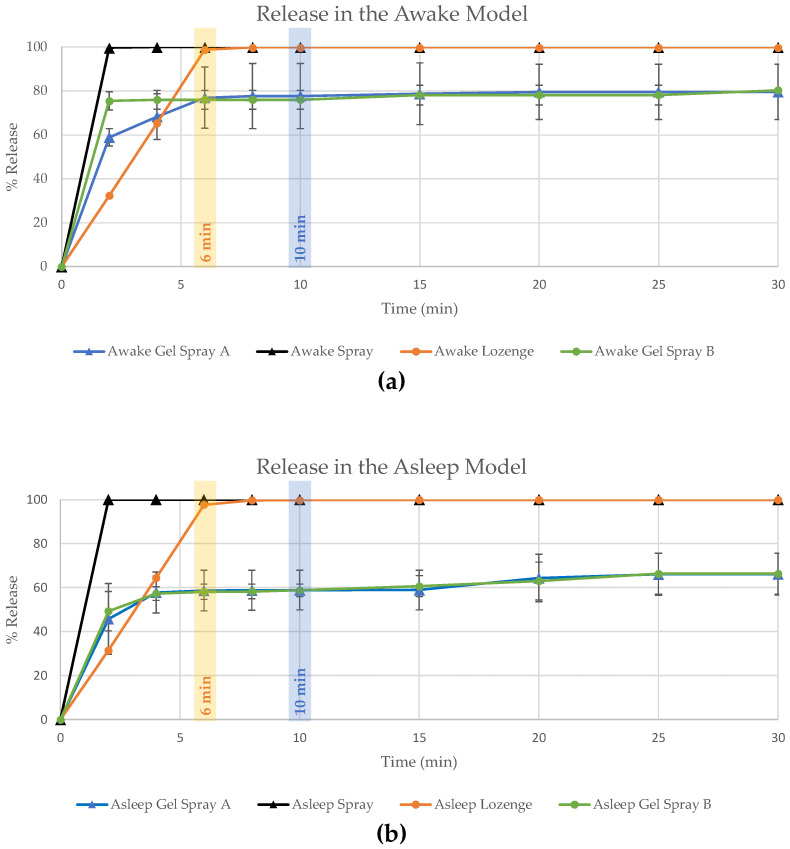
In vitro release of flurbiprofen from different formulations. Percentage released (of applied dose) of flurbiprofen from gel spray A, gel spray B, spray solution and solubilised lozenge after different time periods of rinsing over 30 min, determined according to the Awake (45° incline, (**a**)) and Asleep (30° incline, (**b**)) models.

The different flurbiprofen formulations were investigated using IVOR as a model to mimic bioadhesion in the throat but based on the use of inert membrane (Figure 1). Flurbiprofen from the spray gel formulation adhered to the membrane for the full duration of the experiment (30 min), with 20 and 40% residual amounts flurbiprofen dose in the Awake and Asleep model, respectively. In contrast, flurbiprofen administered via spray solution or solubilised lozenge was nearly completely rinsed from the membrane after two minutes (spray) or eight minutes (lozenge), regardless of whether elution was performed in the Awake or Asleep model. After 6–10 min, release of all formulations in both models apparently reached a plateau, indicating that the most indicative time interval to observe differences in performance of the different formulations seems to be this time interval. In addition, the spray gel formulation not only exhibits better adhesion to the membrane, but also exhibits longer retention in the Asleep model, most probably for mechanical reasons; i.e., lower angle and reduced flow.

Similarly, a higher apparent retention was observed for the lozenge in comparison to the spray solution, probably mainly due to the continuous application for 6 min (to simulate the sucking of the lozenge) during the initial application phase, after which flurbiprofen was rinsed away by blank saliva (2 mL/min).

### 3.2. Rheological Tests

**Figure 2:**
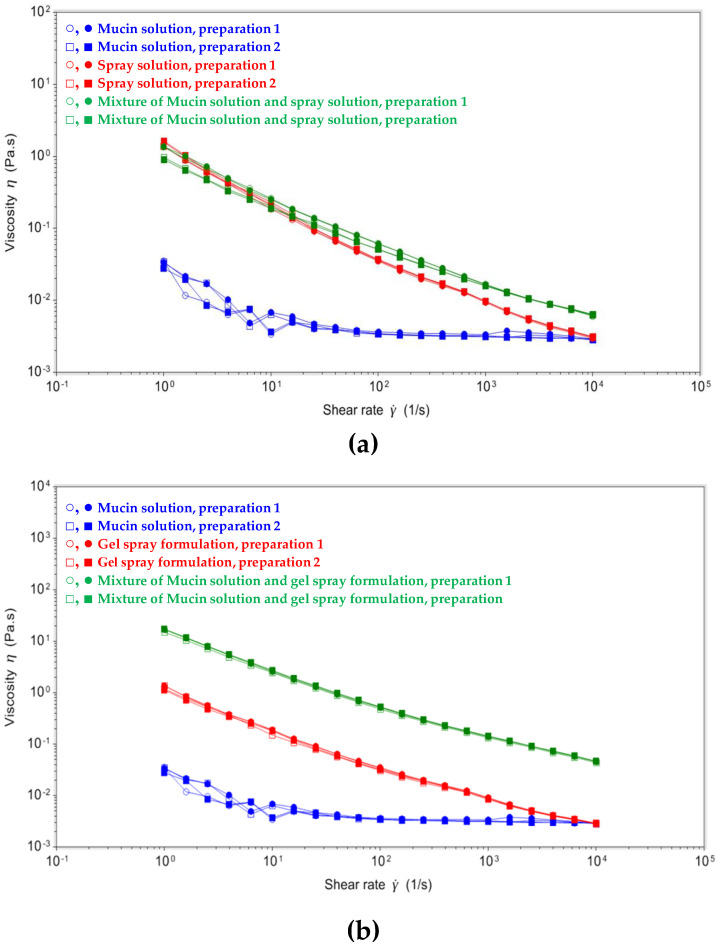
The shear-dependent viscosity profiles of flurbiprofen spray and gel spray, and their mixtures with the mucin control solution. (**a**) The mucin control solution, the flurbiprofen spray and the mixture of both; (**b**) The mucin control solution, the flurbiprofen gel spray and the mixture of both. Shown are results of two independent preparations (circles or squares) each in duplicate measurements with respective standard deviations (plain or empty symbols).

To investigate the influence of the interaction with the biological matrix on bioadhesion at the site of action, the viscosity of the spray solution and the gel spray formulation was assessed in the presence and absence of mucin in rheological tests. A low variability was observed in those measurements (two replicates for the two preparations of each formulation leading to four measurements by formulation). The high viscosity of the spray gel formulation was further increased by interaction with mucin, as observed in the shear dependent viscosity profile (Figure 2). When the spray gel formulation was mixed with a solution containing 3% mucin, the viscosity increased over the whole range of shear rates tested (10^0^–10^4^ s^−1^, Figure 2b). In contrast, when the 3% mucin solution was mixed with the spray solution, no increase in viscosity was observed (Figure 2a). This increase in viscosity of the gel/mucin mixture is attributed to the interaction between the mucin and gel, indicating the mucoadhesive properties of the gel spray formulation.

### 3.3. Ex Vivo and In Vivo Human Data Correlation

**Figure 3:**
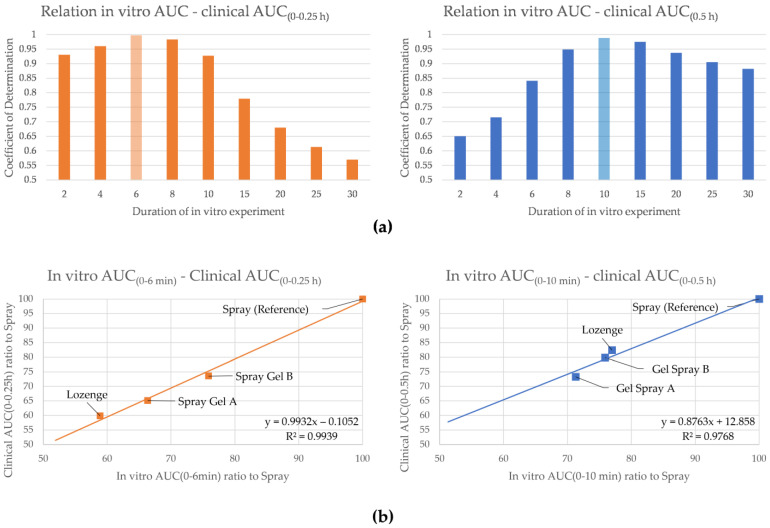
Relationship between clinical AUCs and the AUCs in the in vitro release experiment (Awake model only). Ratio of the Gel Spray A, Gel Spray B and the Lozenge were calculated using the Spray as a reference. (**a**) Relationships were calculated for each time point in the in vitro experiment and the clinical AUC_(0–0.25h)_ (**left**) and the clinical AUC_(0–0.5h)_ (**right**) by linear regression. The coefficient of determination most closely to one is shaded (6 min or 10 min). (**b**) Plots showing the linear regression with best coefficient of determination as determined in (**a**).

As presented in Figure 3, clinical AUC_(0–0.25 h)_ values exhibited a good relationship with in vitro data from 2 to 10 min; in contrast, clinical AUC_(0–0.50 h)_ values exhibited a good relationship with later in vitro data (8 to 15 min). For the clinical AUC_(0–0.25 h)_, which was shown to be the most indicative in terms of predicting the clinical onset of action, the best coefficient of determination was found when using in vitro AUCs for the different formulations at 6 min of the release experiment (Figure 3a, left). For the clinical AUC_(0–0.5 h)_, the best fit was found when comparing with the in vitro AUCs at 10 min (Figure 3a, right). Both coefficients of determination were close to 1, which indicates a link between in vitro release data and clinical AUC data, with the coefficient being closest to 1 when using clinical AUC_(0–0.25 h)_ with in vitro AUCs for 6 min and clinical AUC_(0–0.5 h)_ and in vitro AUCs for 10 min. In the latter case, the ratios of the different formulations approach the reference value, with the lozenge formulation showing the greatest shift (Figure 2b).

Taken together, the relationship of the in vitro and clinical AUCs thus supports the in vivo relevance of IVRT for the characterisation of formulation properties and local absorption, with an in vitro AUC_(0–6 min)_ and a clinical AUC_(0–0.25 h)_, being in line with the finding that clinical AUC_(0–0.25 h)_ shows the best correlation for the clinical onset of action.

As expected, the results for the lozenge move closer to those for the liquid spray (left vs. right panel) after the end of the application period as both formulations are washed away by saliva.

## 4. Discussion

Most modern topical drug products are classified as special’ or complex, terms which describe the non-conventional characteristics of the active entity, delivered from a particular vehicle at a specific site, interacting with a variety of molecules and structures. In vitro tests became the pillar of a new approach frequently described as “extended pharmaceutical equivalence” or “stepwise approach” in the development of generic formulations. The stepwise approach was first introduced by the EMA in 2009 in the guideline for orally inhaled products [21] (although not named in this way), and also in the scientific literature [22], and was recently reinforced in the EMA guideline on LALAs in the GI tract [1]. More recently, both EMA and FDA described ‘extended pharmaceutical equivalence’ (also termed the Q1-Q2-Q3 approach) in draft guidelines/guidances [9,23,24]. For further tests such as in vitro release and in vitro permeation tests, EMA refers to the Draft guideline on quality and equivalence of topical products [9], while FDA describes the approach in two separate documents [25,26].

For topical products the Q1 and Q2 steps refer to comparisons of formulation composition, whereas the Q3 step is an assessment of the physicochemical characteristics and parameters that could influence release and/or adherence of the formulation, for example rheology as a measure of the microstructure of the formulation. After this first set of analyses the similarity of the formulations is assessed through in vitro release tests (IVRT) and in vitro permeation tests (IVPT). If these are not conclusive, in vivo therapeutic equivalence clinical tests could be conducted unless validated pharmacodynamic tests exist (e.g., vasoconstriction test for corticosteroids) [27]. A similar stepwise approach could be used for LALA products acting in the GI tract, where direct in vitro surrogates cannot be used.

The assessment of physical and structural properties through in vitro tests such as rheology, is of key importance for locally applied products and could be considered as a main part of the process as it investigates the inner interactions of the vehicle, the relationship between the active entity and the excipients in a matrix, as well as the interaction between the components of the vehicle and biological components lining the barrier. These interactions are critical for successful delivery of the active substance towards the biological barrier. For a semisolid matrix like a gel spray, the method and means of administration may alter the arrangement of the matter and the resulting resistance that the vehicle expresses for the delivery of the drug. In the example of a gel spray, the device and dosing procedure generate shearing forces which significantly change the vehicle properties before application onto the biological barrier. The resulting changes in viscosity which may affect adherence to the biological barrier can be investigated by rheological methods, including the interaction with biological components like saliva or mucus. Finally, the delivery towards the site of action and the penetration through the biological barrier may also be impacted by the interaction of the active ingredient, components of the vehicle and the biological components lining the barrier. These characteristics can be investigated generally in in vitro adhesion and retention tests on artificial membranes and, more specifically for local administration in the throat, through in vitro permeation tests using human pharynx tissue. Those tests could be seen as the corresponding test to IVRT and IVPT used for topical formulations but adapted to the specificities of the throat administration.

The rheological test assesses the behavior of a formulation and is sensitive to detect formulation modifications which result in changes in the thickness and viscosity of a solution. This test can be considered as a valuable tool to investigate possible retention of the gel. The modification of the setting by pre-incubating the sample with a mucin solution also allows exploration of the mucoadhesive properties of a formulation as proposed by Madsen [28]. This analysis identifies rheological changes that could indicate synergistic interactions that have developed between the test samples and mucin solutions. The increase in the viscosity of the mixture of mucin and the test product is due to interactions between these components and is indicative of mucoadhesive properties for the formulation.

The IVOR model used in the experiments conducted with flurbiprofen formulations was able to discriminate between the formulations under the applied experimental conditions. This test was able to show differences that were not revealed by rheology, due to the particular setup and sensitivity. For example, it was shown that the lozenge had a better retention than the solution spray as confirmed by in vivo data, where initial AUC is lower due to a smaller initial absorption due to a lower amount of flurbiprofen being presented in the throat, 5–6 min sucking as a mean for lozenges were observed in vivo [8]). That denotes the importance of the parameter setting of the experiment that must be in relation with physiology. As presented by Cardot [8] the release behavior of the lozenge must be studied in vitro first to demonstrate the release mechanism and its homogeneity vs. time depending solely on the surface in contact with saliva and the sucking force. This release is not a zero-order process but follows a kinetic described by the decrease of the surface exposed to saliva and the consistency of the lozenge. It is important to highlight that when designing and setting up the IVOR test, the physiological and in vivo drug application conditions need to be taken into account, especially when comparing different pharmaceutical forms. For example, selection of the volume of the lozenge solution should result in concentrations that are likely to be observed in vivo.

The IVOR results were linked to the observed in vivo studies [11]. In order to scale the results between studies and between in vitro and in vivo, the AUC ratio using spray as reference was used. Based on clinical studies, the distinction between flurbiprofen lozenge, spray gel and spray solution is mainly visible at early time points [11], with the early exposure (expressed as AUC_(0–0.25 h)_ and AUC_(0–0.5 h)_) showing a significant correlation to the clinical onset of action (time to clinically relevant pain relief). Up to 30 min, the higher the ratio of the AUC (with the spray solution formulation as reference), the shorter is the time to onset of action. For example, for the lozenge which exhibits ratios of 59.86% and 84.12% vs. the spray solution at 0.25 h and 0.5h, respectively, the onset of action was at 26 min compared to the spray solution which had an onset of action at 20 min. In contrast, the formulations, including sprays (simple solution and gel spray) and lozenges all exhibit a very similar extent of absorption as expressed by AUC_0-t_ and rate of absorption as expressed by C_max_ values (bioequivalence criteria, defined as 90% CI within the standard range of 80–125%) [11] and thus such studies do not provide sufficient differentiation of presented locally applied flurbiprofen formulations. In keeping with these general bioavailability findings, the clinical observations made in therapeutic non-inferiority studies also demonstrated similar efficacy for these respective formulations [29,30].

In vitro permeation tests were performed by means of an in vitro pharynx permeation/penetration test with human ex vivo pharynx tissue using a Frantz cell system equipped with a micro-adapter for solubilised lozenge and spray solution. The methodology and results are described by Turner [12,13]. In brief, the different formulations were applied to the pharynx tissue on donor side of the chamber for 60 min, and the penetrated flurbiprofen was recovered on the other side of the tissue, in the fluid of the receptor chamber. At the end of the study, cotton swabs were taken from the donor chamber (tissue surface), and the extent of flurbiprofen penetration into the human pharynx tissue was assessed based on analysis of pharynx tissue slices. The study indicated that the relative quantity at the surface of the pharynx tissue for spray solution is slightly higher than for lozenge, however, the evaluation of the observed difference is limited: due to the experimental conditions selected, the average dose of the two products compared was very different; this did not allow for quantitative evaluation due to the sensitivity of analytical method used. The extent of flurbiprofen penetration into human pharynx tissue was in general comparable between spray solution and lozenge formulations despite the differences in the drug concentrations and total drug dose exposure between the two formulations. The majority of the doses applied did not penetrate beyond the depth of about 2 mm, and consequently, only traces of flurbiprofen (<1% of total dose) penetrated through the tissue and were recovered in the receptor fluid. In summary, the differences observed in the penetration fraction (receptor fluid and within the tissues) are very small and therefore the differences in the formulations studied do not significantly impact the penetration of flurbiprofen in the pharynx tissue. This was confirmed by the observation that no relevant differences were found between the two formulations with respect to the depth of flurbiprofen permeation in pharyngeal tissue. However, the results of this study highlight the difficulty of balancing physiological conditions with analytical settings, especially for the solid dosage form and spray. For the lozenge, the dissolution in liquid to mimic the possible concentration observed in vivo in contact to the throat could lead to difficulty in the accurate assessment of penetrated fraction over time, unless very sensitive analytical methods are used. It must also be noted that this test focuses on the possible penetration and is not adapted to throat administration as the drug will not remain in tight contact with the tissue surface in high concentrations for 60 min due to gravity (throat is not horizontal in contrast to Frantz cell membrane) and continuous washing due to saliva deglutition (0.5 to 7 mL/min depending on the stimulation as a mean 1.5 mL/min of saliva produced correspond as a mean to 90 mL/h) [16,18]. That denotes that classical IVRT and IVPT test might not be optimal to evaluate throat formulations.

Our IVOR model in contrast tried to mimic those two factors: inclination of the throat and continuous washing. The interest of this test is highlighted by the relation established between the in vitro release as assessed by the Awake model and expressed as AUCs of the various formulations in vitro and the results observed in vivo as previously reported by Perlik et al. [11]. The results indicate that higher retention (slower release) corresponds with smaller regional absorption in vivo, expressed as clinical AUC_(0–0.25 h)_ and AUC_(0–0.5 h)_. This is of particular importance as the early onset of clinical action, linked with local absorption, is detectable also based on IVRT.

The assessment of the feasibility of replacing preliminary bioavailability or clinical studies with a series of in vitro tests requires consideration of the advantages and disadvantages of each approach. For example, bioavailability study assesses the plasma concentration and not the concentrations at the site of action. Plasma concentration are of importance in case of possible side effects and in this case the test formulation must be lower or equal to the reference formulation. However plasma concentration might not reflect concentration at the site of action. It is well known that concentration at the site of action could be greater than in the plasma concentrations for topical drug that being the case for NSAIDs. For example, from the skin and underlying tissue, diclofenac preferentially distributes and persists in deep inflamed tissues [31].

Clinical studies have the great advantage of evaluation in a real therapeutic setting. This, however, introduces the natural variability of human individuals, which may not be the most suitable environment for comparing different formulations of the same drug substance. For such evaluations, and in particular for LALA products, the question is whether reliable conclusion can be made based on variable clinical data, and more importantly, whether the outcome of a clinical study accurately reflects the quality and performance of the drug product or only the variability of interaction between the product and the particular subjects and their environment (patient baseline characteristics, study team, site, country, region, culture, etc.). Moreover, the question arises whether a clinical study for a LALA drug product is sufficiently sensitive for the formulation effects and strength, considering the complexity of the delivery context. In addition, in many cases the drug administered locally and acting locally exhibits few side effects, and so the doses applied could be higher than needed and can be dispensed less accurately. In keeping with this, spray delivery is at ±15% according to EP and USP monographs and guidelines [32,33,34,35].

In this context, well designed in vitro tests in a setting which tries to reproduce the physiological key factors might be more rapid, sensitive and discriminating than in vivo tests, which could subsequently be limited to specific cases only.

## 5. Conclusions

The results presented in the current paper show that in vitro tests, including assessment of rheological properties and in vitro release using the IVOR model, allow the characterisation of various aspects of locally applied, locally acting formulations which are related to in vivo results. Those techniques were applied successfully to lozenges, spray solutions and altered increased viscosity solutions (e.g., gel sprays).

Even in vitro tests could not replace all the in vivo studies; the key advantages of the in vitro tests are their sensitivity, reliability and reproducibility. Designing a relevant in vitro test requires adequate evaluation of the complexity of the drug substance, drug product, dosing conditions and delivery processes. These steps may be assessed individually or in aggregate using the previously described tests. Their combined results reflect not only the performance of the dosage form in relation to a specific therapeutic outcome, but also the risks of not achieving this particular level of performance. Moreover, in vitro testing establishes a science-based bridge between routine quality control and therapeutic outcome, leading to adequate finished product specifications by selection of appropriate parameters and acceptance ranges. This also allows for flexibility in future optimisation of the composition and manufacturing process, as well as reduced costs for research and development and, consequently, increased availability and affordability of these products for patients.

## Data Availability

Data are available on request for scientific reasons.
